# Atrophe Pseudarthrose des Sternums – ein Fallbericht orientiert am Non Union Scoring System

**DOI:** 10.1007/s00113-022-01144-5

**Published:** 2022-02-08

**Authors:** Benedikt J. Braun, Steven C. Herath, Mika F. R. Rollmann, Maximilian M. Menger, Heiko Baumgartner, Marie Reumann, Tina Histing

**Affiliations:** Klinik für Unfall- und Wiederherstellungschirurgie an der Eberhard Karls Universität Tübingen, BG Klinik Tübingen, Schnarrenbergstr. 95, 72076 Tübingen, Deutschland

**Keywords:** Knöcherne Fehlheilung, Brustbein, Winkelstabile Sternumplatten, NUSS-Score, Cutibacterium Acnis, Failed Bone Healing, Breastbone, Locking sternum plates, NUSS score, Cutibacterium acnis

## Abstract

Pseudarthrosen nach Sternotomien sind rar und meist gut im Rahmen operativer Revisionen zu behandeln. Bei fehlgeschlagenen Revisionen ist ein differenziertes Vorgehen notwendig, das sowohl die biologischen wie auch biomechanischen Rahmenbedingungen berücksichtigt. Es wird über den Fall einer therapierefraktären atrophen Pseudarthrose nach auswärtig erfolgloser Revision berichtet. Diese wurde durch ein Therapievorgehen, orientiert an den Kriterien des Non Union Scoring System (NUSS), erfolgreich behandelt. Die Versorgungsstrategie wie auch der erfolgreiche Behandlungsverlauf werden dargestellt.

## Abstract (en)

Nonunions after median sternotomy are rare and usually respond well to surgical treatment. Recalcitrant nonunions despite surgical treatment require a comprehensive mechanical and biological treatment strategy to achieve an adequate functional result for the patient. We demonstrate the case of a 4-year recalcitrant atrophic nonunion. Through a surgical approach guided by the criteria of the nonunion scoring system (NUSS) successful healing was achieved. Our strategy as well as the treatment course are presented.

Die mediane Sternotomie stellt einen häufigen Zugangsweg für große Herz-/thoraxchirurgische Eingriffe dar und ist mit einer insgesamt niedrigen Pseudarthroserate vergesellschaftet [6]. Verschiedene konservative [6, 7] wie auch operative Revisionsmöglichkeiten wurden in der Literatur beschrieben [11]. Dabei werden gute radiologische Ausheilungsergebnisse wie auch funktionelle Resultate berichtet. Persistierende Pseudarthrosen nach Sternotomie sind selten, und nur wenige Fallberichte existieren. Die aktuelle Kasuistik stellt den Fall einer solchen Pseudarthrose vor und beschreibt unser kombiniertes mechanisches und biologisches Vorgehen, orientiert am Non Union Scoring System (NUSS) [4].

## Falldarstellung

### Anamnese

**Figure 1:**
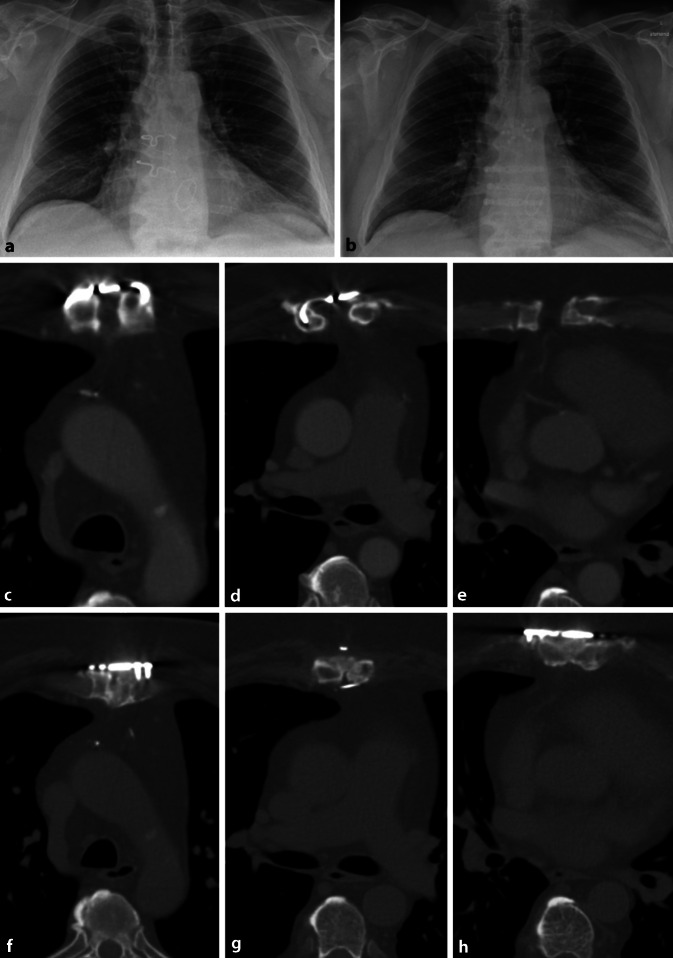

Ein 57-jähriger Patient stellte sich zur Einholung einer Zweitmeinung bei therapierefraktärer Pseudarthrose des Sternums in unserer Pseudarthrosenspezialsprechstunde vor. Vorausgegangen war ein Mitralklappenersatz im Jahr 2017 über eine vollständige mediane Sternotomie, die mit Draht-Cerclagen verschlossen wurde. Hier zeigte sich 6 Monate postoperativ eine Dehiszenz der Sternumhälften bei ausbleibender Heilung, sodass die Diagnose einer atrophen Pseudarthrose des Sternums gestellt wurde. Bei ausgeprägter Beschwerdesymptomatik mit persistierendem Dauerschmerz, nichtmöglicher Seitlagerung sowie einschießenden Schmerzen bei tiefem Atmen, Husten und Niesen wurde durch auswärtige thoraxchirurgische Kollegen die Pseudarthrose durch Anlage von 2 Klammer-Cerclagen operativ revidiert (Abb. 1a,b). Im Verlauf kam es zu einer persistierenden Pseudarthrose mit ausgeprägten Schmerzen beim tiefen Atmen, Husten/Niesen, Heben sowie in Seitenlage. Bei fehlender Beschwerdebesserung und Ausheilung unter konservativen Maßnahmen mit konfektionierten Kompressionsverbänden und Vitamin-D-Substitution wurde auswärtig 3 Jahre nach dem Ersteingriff erneut eine Revision geplant, in deren Rahmen eine erneute Cerclagen-Versorgung durchgeführt werden sollte. Hierauf erfolgte durch den Hausarzt die Überweisung zur Zweitmeinung an unsere Spezialsprechstunde.

### Befund

Es bestand eine ausgeprägte Druckschmerzhaftigkeit mit tastbarer Dehiszenz der beiden Sternumhälften mit anteroposteriorem und seitlichem Thoraxkompressionsschmerz. Die Zugangsnarbe zeigte sich hypertroph, aber regelrecht verheilt, ohne lokale Infektzeichen. Die infektiologischen, laborchemischen Parameter (Leukozyten, CRP) waren ebenfalls unauffällig. Die zur Vorstellung durchgeführte CT-Diagnostik des Thorax zeigte eine atrophe Pseudarthrose (Abb. 1c–e) mit abgerundeten Sternumenden, ohne Kallusbildung und mit einer Dehiszenz von bis zu 18 mm. Die verbliebenen Sternumhälften zeigten eine durchschnittliche Breite von 8–10 mm. An grundsätzlichen Risikofaktoren bestanden bei unserem Patienten eine Adipositas (BMI 40,6 kg/m^2^), ein nichtinsulinpflichtiger Diabetes mellitus mit hyperglykämischen Werten (151 mg/dl), sowohl im Rahmen der Erstvorstellung als auch im Behandlungsverlauf, sowie eine intermittierende Einnahme von nichtsteroidalen Antirheumatika. Spezifische Risikofaktoren für eine Sternumdehiszenz bestanden durch die bereits erfolgte operative Revision, den hohen BMI, das männliche Geschlecht des Patienten sowie den nichtinsulinpflichtigen Diabetes mellitus [10].

## Diagnose

Atrophe Pseudarthrose nach medianer Sternotomie mit einem NUSS-Score von 36 (Tab. 1).

**Table 1:** 

	Punkte
*Knochenqualität*	
Gut	*0*
Moderat (milde Osteoporose)	1
Schlecht (schwere Osteoporose/Knochenverlust)	2
Sehr schlecht (nekrotischer Knochen, avaskulär/septisch)	3
*Ursprüngliche Verletzung (Gustilo-Anderson-Klassifikation)*	
Geschlossen	*0*
1° offen	1
2–3a° offen	3
3b–c° offen	5
*Anzahl vorausgegangener Interventionen*	
Keine	1
≤ 2	*2*
≤ 4	3
> 4	4
*Invasivität vorausgegangener Interventionen*	
Minimal-invasiv (geschl. Therapie; K‑Drähte; Schrauben)	0
Intramedulläre Stabilisierung	1
Extramedulläre, aber interne Stabilisierung	*2*
Jegliche Osteosynthese, die ein Knochentransplantat beinhaltet	3
*Adäquanz des Primäreingriffes*	
Inadäquate Stabilität	0
Adäquate Stabilität	*1*
*Weber-und-Cech-Einteilung*	
Hypertroph	1
Oligotroph	3
Atroph	*5*
*Stellung des Knochens*	
Nichtanatomisches Alignment	0
Anatomisches Alignment	*1*
*Defektgröße*	
Bis 1 cm	2
1–3 cm	*3*
Größer 3 cm	5
*Weichteilstatus*	
Intakt	0
Unauffällige postoperative Heilung, minimale Narbenbildung	*2*
Vorausgegangene Weichteildefektbehandlungen (Hautverlust, mehrere Inzisionen, lokaler Lappen, Kompartmentsyndrom)	3
Vorausgegangene komplexe Weichteilbehandlung (freier Lappen)	4
Schlechte Vaskularität (keine distalen Pulse, schlechter kapillärer Refill, venöse Insuffizienz)	5
Vorhandensein einer Hautläsion/Defekt (Ulkus, Sinus, frei liegender Knochen oder Implantate)	6
*ASA-Klassifikation, Patient*	
ASA 1/2	*0*
ASA 3/4	1
*Diabetes*	
Keiner	0
Ja – gute Blutzuckerkontrolle (HbA_1c_ < 10)	*1*
Ja – schlechte Blutzuckerkontrolle (HbA_1c_ > 10)	2
*Labor (Pro Laborwert 1 Punkt möglich)*	
Leukozyten > 12.000/mm^3^	1
Blutkörperchensenkungsgeschwindigkeit > 20 mm	1
CRP > 20 mg/l	1
*Klinischer Infektstatus*	
Keiner	*0*
Vorausgegangene Infektion oder vermutete Infektion	1
Septischer Befund	4
*Medikamenteneinnahme (Pro Gruppe 1 Punkt möglich)*	
Kortikosteroide (Einnahme über > 5 Tage während der letzten 3 Monate)	1
NSAR (Einnahme über > 5 Tage während der letzten 3 Monate)	*1*
*Nikotinkonsum (jeglicher Konsum innerhalb der letzten 6 Monate)*	
Nein	*0*
Ja	5

Gezeigt ist die Matrix zur Berechnung des NUSS Score nach Calori et al. [3]. Zur Berechnung des Gesamtscores werden die berechneten Punkte verdoppelt. Abgeleitet sehen Calori et al. bei Punktzahlen bis 25 eine rein mechanische Genese der Pseudarthrose, bis 50 Punkte eine kombinierte biologisch mechanische Genese, die einer Korrektur beider Faktoren bedarf, bei 51 bis 75 Punkten eine ausgeprägte kombinierte Genese, die neben der operativen Ausräumung und Stabilisierung zusätzlich eine biologische „Polytherapie“, bestehend aus u  a. autologer Spongiosa, freien Transplantaten oder biotechnologischen Produkten benötigt. Bei über 75 Punkten wird eine Therapie, angepasst an die patientenindividuellen Verhältnisse beschrieben, die bei Extremitätenpseudarthrosen bis hin zur Amputation gehen kann [2]. Die in unserem Fall vergebenen Punktwerte sind kursiviert

## Therapie und Verlauf

In der Zusammenschau der radiologischen Bildgebung, der Risikokonstellation des Patienten sowie des klinisch, laborchemisch unauffälligen Befundes wurde analog der NUSS-Einstufung ein einzeitiges Vorgehen mit mechanischer Stabilisierung und biologischer Augmentation mittels autologer Beckenkammspongiosa gewählt.

**Figure 2:**
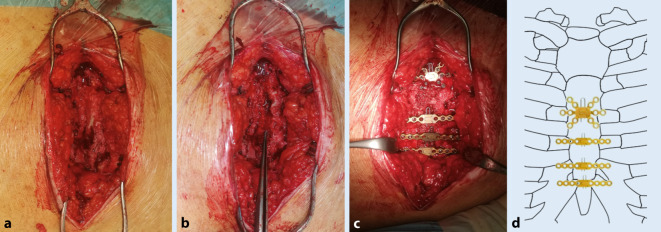

Die Operation erfolgte unter Allgemeinanästhesie in Rückenlagerung auf dem Carbontisch. Hier empfiehlt sich die Auslagerung der Arme zur rasch möglichen, intraoperativen Anlage einer Thoraxdrainage, falls benötigt. Die ehemalige Inzision wurde unter Ausschneidung der Wundränder wiedereröffnet. Nach der Resektion des Narbengewebes wurde das Sternum vom Übergang des Manubriums zum Corpus bis zum distalen Ende vollständig epiperiostal freigelegt. Nach Entfernung der einliegenden Klammer-Cerclagen erfolgte ein radikales Débridement des fibrösen Gewebes im Pseudarthrosenspalt wie auch der sklerosierten Ränder des Sternums zu beiden Seiten (Abb. 2a,b). Zum Abschluss des Débridements zeigte sich zu beiden Seiten vitaler, spongiöser Knochen über die gesamte Länge der beiden Sternumhälften unter Schonung des Mediastinums. Aus dem débridierten Material wurden insgesamt 5 Proben zur mikrobiologischen Untersuchung eingeschickt. Parallel wurde mit einem zweiten sterilen Tischset autologe Beckenkammspongiosa entnommen. Diese wurde nach ausgiebiger Spülung des Befundes mit Ringer-Lösung auf der gesamten Strecke des Sternums angelagert und das Sternum anschließend mit 2 Repositionszangen proximal und distal komprimiert und verschlossen. Die Spongiosa wurde zusätzlich verdichtet und auch in reponiertem Zustand nachträglich weiterangelagert. Nach radiologischer Kontrolle in a.-p.- und seitlichem Strahlengang einer regelrechten Reposition erfolgte Positionierung von vier 2,4-mm-Sternumplatten mit Sicherungsstift (Sternum-Fixationssystem aus Titan, Fa. Synthes, Umkirch, Deutschland). Eine sternförmige Platte, 6 × 6-Loch, wurde im kranialen Anteil des Corpus sterni platziert, 3 weitere einreihige Platten wurden auf den kaudalen Costae verae platziert und gemäß der präoperativen CT-Messungen mit selbstbohrenden, winkelstabilen Schrauben besetzt (Abb. 2c). Hierunter zeigte sich der Befund auch nach Abnahme der Repositionszangen stabil. Nach Abschlussbildgebung in a.-p.- und seitlichem Strahlengang erfolgte der schichtweise Wundverschluss unter Bedeckung des Sternums mittels Pektoralisverschluss. Der Patient konnte mit kardiopulmonal stabilem Befund postoperativ auf die Normalstation übernommen werden.

Bereits unmittelbar postoperativ gab der Patient an, den Stabilitätsunterschied im Sternum zu verspüren. Die Nachbehandlung erfolgte unter Vermeidung grober Hebe- und Abstützbelastung sowie Limitation der Anteversion und Abduktion der Schulter bis 70° für 6 Wochen. Darüber hinaus wurde der Patient angewiesen, in diesem Zeitraum zum Husten ein Kissen vor den Thorax zu pressen. Nach 5 Tagen konnte der Patient mit reizlosen Wundverhältnissen, unauffälliger Laborchemie eigenständig voll mobilisiert nach Hause entlassen werden. In der 14-tägigen Bebrütung der intraoperativ entnommenen mikrobiologischen Proben ließ sich in 4 aus 5 Proben ein *Cutibacterium acnes* nachweisen, welches aber aufgrund nur eingeschränkten Kulturwachstums nicht weiter differenziert oder resistenzausgetestet werden konnte. Bei weiterhin unauffälligem klinisch, laborchemischem Befund, intraoperativ radikalem Débridement und vollständiger Beschwerdefreiheit des Patienten erfolgten diesbezüglich keine weiteren operativen Maßnahmen. Nach dem Befundeingang wurde für 4 Wochen eine antiinfektive Therapie mit Amoxicillin durchgeführt. Nach 6 Wochen und 3 Monaten erfolgte eine klinisch-radiologische Verlaufskontrolle. Die Weichteile im Narbenareal des Sternums zeigten sich reizlos und unauffällig. In der CT-Verlaufskontrolle nach 3 Monaten ließ sich eine langstreckige Durchbauung des Befundes (Abb. 1f–h) mit unveränderter Implantatlage nachweisen. Der Patient zeigte sich vollständig beschwerdefrei mit freier Schulterfunktion, ohne Einschränkungen bei Atmen, Husten oder Niesen, nun erneut möglichem Seitenschlaf und vollständig in das Berufsleben als Außendienstmitarbeiter wieder eingegliedert.

## Diskussion

Pseudarthrosen nach medianer Sternotomie sind selten. In einer retrospektiven Serie aus über 12.000 Sternotomien konnten aus der Arbeitsgruppe um John Conte lediglich 48 Pseudarthrosen identifiziert werden [11]. Die Therapie der Wahl war hier in der überwiegenden Zahl der Fälle eine erneute Cerclagen-Versorgung und Stabilisierung des Sternums ohne weitere biologische Maßnahmen. Wenige Fallberichte über therapierefraktäre Pseudarthrosen mit vorangegangener Stabilisierung existieren und empfehlen pauschal eine autologe Spongiosaplastik zusätzlich zur mechanischen Stabilisierung [2]. In unserer Erfahrung aus einer großen Pseudarthrosenspezialsprechstunde hat sich gerade bei vermeintlich therapierefraktären Pseudarthrosen ein systematisches Vorgehen unter Berücksichtigung sowohl der mechanischen wie auch der biologischen Verhältnisse in der Pseudarthrose bewährt. Eine simple mechanische Stabilisierung, wie sie in unserem Fall auswärtig als erneute Revision geplant war, hätte in diesem Fall mutmaßlich ein weniger zuverlässiges Ausheilungsergebnis gezeigt. Bei atrophischen Verhältnissen mit großer Dehiszenz und bereits vollständig sklerosierten Frakturrändern ist eine reine mechanische Korrektur wenig vielversprechend, wie auch von Scoring-Systemen wie dem NUSS empfohlen (Tab. 1). Dies bestätigt, dass diese Scoring-Systeme ein wichtiger Anhalt für die notwendigen therapeutischen Schritte sein können und das Bewusstsein für weitere biologische Maßnahmen schärfen [3]. Der NUSS-Score kommt im Rahmen unserer Sprechstunde regelmäßig bei differenzialtherapeutischen Überlegungen zum Einsatz und wurde im Rahmen dieser Behandlung auch erstmalig zur Therapie einer Sternumpseudarthrose herangezogen. Publikationen zu diesem Score sind den Autoren zur Entität der Pseudarthrose am Sternum bisher nicht bekannt. Das Score-Ergebnis von 36 in unserem Fall legt eine zusätzliche biologische Behandlung, wie sie in unserer Therapie mittels autologer Spongiosaanlagerung erfolgt ist, nahe. Eine winkelstabile Plattenversorgung wurde gewählt, da wir hier eine größtmögliche Stabilität und Verankerungsmöglichkeit im wiederholt voroperierten Sternum sahen. Wenngleich bezüglich der Versorgung mit diesen Plattensystemen und selbstbohrenden Schrauben eine abschließende wissenschaftliche Bewertung basierend auf sowohl biomechanischen wie auch systematischen klinischen Studien aussteht, sehen wir für diese Stabilisierungsform vor dem Hintergrund des Risikoprofils unseres Patienten Vorteile in der aktuellen Literatur zu Revisionen am Sternum [9, 10].

Der Keimnachweis in einem Teil der intraoperativen Proben zeigt darüber hinaus das Dilemma der latenten Infektion. Gerade bei nur eingeschränktem Kulturwachstum ist immer auch eine Verunreinigung der Proben denkbar, allerdings legen aktuelle Studien nahe, dass ein größerer Anteil vermeintlich aseptischer Pseudarthrosen doch durch eine latente Keimbesiedelung mitbedingt sein kann [8]. Bei unserer Behandlung mit Nachweis in 4 aus 5 Proben ist eine Kontamination entsprechend unwahrscheinlich. Hier zeigt sich noch einmal eindrücklich die Wichtigkeit einer ausgedehnten Probennahme. Ergänzend kann hier auch die Gewinnung einer pathologischen Probe, die in unserem Fall leider nicht erfolgt ist, die Interpretation der Befunde vereinfachen. Bei makroskopisch unauffälligem Operationssitus erlaubt ein radikales Débridement, wie es auch im vorliegenden Fall eingesetzt wurde, eine auch einzeitige Behandlung, gerade wenn präoperativ und intraoperativ keinerlei Anzeichen und Risikofaktoren für ein Infektionsgeschehen vorlagen. Das gezeigte Keimspektrum ist bei anderen thoraxnahen Frakturen und Pseudarthrosen ebenfalls häufig und die klinische Signifikanz dieser Befund nicht abschließend geklärt [5]. Eine entsprechende Erfahrung wie auch die gemeinsame, patientenindividuelle Behandlung mit Kollegen aus der septischen Chirurgie an Zentren, die auf die Behandlung von Pseudarthrosen spezialisiert sind, ist in diesen Fällen nach unserer Meinung vorteilhaft. Aufgrund der unauffälligen laborchemischen wie auch klinischen Befunde vor unserem Revisionseingriff ist präoperativ keine weitergehende Abklärung bezüglich einer etwaigen infizierten Pseudarthrose durch Punktion oder spezialisierte weitere Bildgebung erfolgt [12]. Ebenfalls wurde bei intraoperativ unauffälligem Befund postoperativ keine prophylaktische antiinfektive Therapie durchgeführt. Nachdem die Langzeitbebrütung den Nachweis von *Cutibacterium acnes* zeigte, wurde eine antiinfektive Therapie mit Amoxicillin begonnen und für 4 Wochen bis zur ersten klinisch radiologischen Kontrolle weitergeführt. Hier wurde die Therapie bei weiterhin unauffälligem Verlauf und unauffälliger Laborchemie sowie eingeschränkter Verträglichkeit beim Patienten beendet. Die Empfehlungen zur antiinfektiven Therapiedauer sind in der Literatur kontrovers diskutiert und ohne einheitliche, evidenzbasierte Empfehlung. Abzugrenzen ist die latente Infektion von einer Sternumosteomyelitis, die mit ihrem fulminanten Verlauf ein frühzeitiges, radikales Débridement mit einer zielgerichteten Behandlung erfordert [1].

## Fazit für die Praxis

- Der Fall zeigt, dass zur Behandlung initial therapierefraktärer Pseudarthrosen ein differenziertes Vorgehen unter Berücksichtigung der Stabilität wie auch der Biologie des Befundes notwendig ist.
- Etablierte Scoring-Systeme, wie der NUSS-Score können helfen, die notwendigen Therapiemaßnahmen abzuschätzen.
- Bei der operativen Revision von Sternumpseudarthrosen kann die Verwendung von winkelstabilen Implantaten sinnvoll sein.
- Im Gegensatz zu einer latenten Keimbesiedelung ist die Sternumosteomyelitis aufgrund des erhöhten Risikos einer fulminanten Mediastinitis mit einer Mortalität zwischen 15 und 30 % verbunden und macht ein sofortiges radikales Débridement bis zur Infektberuhigung notwendig.
